# Detecting and grading prostate cancer in radical prostatectomy specimens through deep learning techniques

**DOI:** 10.6061/clinics/2021/e3198

**Published:** 2021-10-20

**Authors:** Petronio Augusto de Souza Melo, Carmen Liane Neubarth Estivallet, Miguel Srougi, William Carlos Nahas, Katia Ramos Moreira Leite

**Affiliations:** Laboratorio de Pesquisa Medica - LIM55, Divisao de Urologia, Faculdade de Medicina FMUSP, Universidade de Sao Paulo, Sao Paulo, SP, BR.

**Keywords:** Prostate Cancer, Deep Learning, Radical Prostatectomy, Prostate Pathology, Artificial Intelligence

## Abstract

**OBJECTIVES:**

This study aims to evaluate the ability of deep learning algorithms to detect and grade prostate cancer (PCa) in radical prostatectomy specimens.

**METHODS:**

We selected 12 whole-slide images of radical prostatectomy specimens. These images were divided into patches, and then, analyzed and annotated. The annotated areas were categorized as follows: stroma, normal glands, and Gleason patterns 3, 4, and 5. Two analyses were performed: i) a categorical image classification method that labels each image as benign or as Gleason 3, Gleason 4, or Gleason 5, and ii) a scanning method in which distinct areas representative of benign and different Gleason patterns are delineated and labeled separately by a pathologist. The Inception v3 Convolutional Neural Network architecture was used in categorical model training, and a Mask Region-based Convolutional Neural Network was used to train the scanning method. After training, we selected three new whole-slide images that were not used during the training to evaluate the model as our test dataset. The analysis results of the images using deep learning algorithms were compared with those obtained by the pathologists.

**RESULTS:**

In the categorical classification method, the trained model obtained a validation accuracy of 94.1% during training; however, the concordance with our expert uropathologists in the test dataset was only 44%. With the image-scanning method, our model demonstrated a validation accuracy of 91.2%. When the test images were used, the concordance between the deep learning method and uropathologists was 89%.

**CONCLUSION:**

Deep learning algorithms have a high potential for use in the diagnosis and grading of PCa. Scanning methods are likely to be superior to simple classification methods.

## INTRODUCTION

The high prevalence and complex management of prostate cancer (PCa) have imposed significant amounts of investment in healthcare systems (1,2). The wide spectrum of aggressiveness of PCa, ranging from an indolent disease that can be managed with surveillance to an aggressive disease with a poor prognosis, necessitates accurate diagnosis and classification. Tumor grading using the Gleason/ISUP score is the main prognostic factor, and together with staging, indicates the choice of treatment and probable outcome (3,4). Histological analysis and Gleason/ISUP grading are currently conducted subjectively by pathologists, and although there are many initiatives aiming to train as many specialists as possible, the number of pathologists is insufficient for dealing with the increasing number and complexity of the actual requirements (5-7).

Among expert uropathologists, the disagreement in determination based on the Gleason score reaches up to 12%; however, this number increases to 50% when considering generalist pathologists (8,9).

In the last few years, the field of knowledge on artificial intelligence has rapidly increased. Machine learning has become prevalent, and is present in many high-tech products, including web search results, speech recognition in smartphones, and video recommendations, among other tasks.

In 2006, Hinton et al. (10) described how to train a machine that is capable of recognizing handwritten digits with high precision (>98%), an approach they called “deep learning.”

Deep learning is a branch of artificial intelligence that processes data and creates patterns for use in decision-making (11). In recent years, researchers have tried to solve the problem of PCa diagnosis and grading using deep learning techniques to overcome the current limitations of human-made diagnoses (12-14).

In this study, we used prostatectomy specimens evaluated by experienced uropathologists to train a deep learning algorithm in the detection and grading of PCa.

## MATERIALS AND METHODS

### Study population, slide digitization, and annotation

We randomly selected 12 whole-slide images of hematoxylin-and-eosin-stained formalin-fixed paraffin-embedded prostatectomy specimens from our database slides. Each analyzed slide belonged to a different patient. These slides were digitized at a magnification of 20× using a Pannoramic Flash II 250 scanner (3DHISTECH Ltd., Budapest, Hungary). The whole slides were segmented into 1,525 image patches with a pixel resolution of 2,000×2,000 using Python 3 (https://www.python.org). These image patches were then analyzed and annotated by two experienced uropathologists (K.R.M.L. and C.L.N.E.). The annotations were initially conducted separately by the pathologists, and all images were shown. When a disagreement occurred in any image, the pathologists discussed the particular image and reached a consensus. Two different analyses and annotations were employed.

– Categorical image classification method: We labeled each image according to the presence or absence of malignancy. Within the cancer images, each image was labeled according to the most prevalent Gleason pattern present on the slide. As the output, each predicted image was classified into one of four patterns: benign, Gleason 3, Gleason 4, or Gleason 5.
– Image scanning method: Using this method, we delineated and annotated specific areas in each image, rather than simply classifying the entire image with a single label. To accomplish this task, we used the co-annotator tool (https://github.com/jsbroks/coco-annotator/). Each annotation belonged to one of five categories: stroma, normal glands, or Gleason pattern 3, 4, or 5.

### Development of deep learning algorithm

In the categorical classification method, we used the Inception v3 Convolutional Neural Network Architecture (https://github.com/machine-learning/Inception-v3-tensorflow) and TensorFlow library (https://www.tensorflow.org) to train the model. The image patches were divided into training and validation datasets. The training dataset is an actual dataset used to train the model. The model observes and learns from these data. Meanwhile, the validation dataset is the sample of data used for frequent evaluations of the model, the hyperparameters of which are turned. The model sees the validation dataset, but never learns from it. Because robust datasets are required for adequate network training, we applied data augmentation on all image patches of our training data: horizontal and vertical flipping, rotation, and zooming.

With the scanning method, the image patches were divided into training and validation data. The model was trained using the Mask Region-based Convolutional Neural Network (Mask R-CNN) (https://github.com/matterport/Mask_RCNN), where the model learns from the delineated areas annotated by the pathologists and generates its own bounding boxes and segmentation masks for each instance of an object in the image.

### Model evaluation

After the model training, we selected three new whole-slide images that were not used in the training, to evaluate the generalization capability of the model. We prepared these images in the same way as with the training images, *i.e.*, using image patches with a pixel resolution of 2000×2000. From them, we randomly chose 100 different image patches for each classification method. All images were evaluated using deep learning algorithms after being read by the uropathologists, and the concordance between the two results was analyzed.

### Ethics

The study was approved by the Institutional Review Board and Ethics Committee, and informed consent was considered unnecessary.

## RESULTS

Using the categorical classification method, 740 images in the benign group and 785 images in the cancer group (251 for Gleason 3, 254 for Gleason 4, and 280 for Gleason 5) were categorized. The images were randomly separated into training (1,220 images) and validation (305 images) data.

With the scanning method, from the 1,525 images, the pathologists made 1,982 annotations, which were divided into 559 normal glands, 535 stroma, 273 Gleason 3, 281 Gleason 4, and 334 Gleason 5 annotations. Likewise, the images were randomly divided into two groups, *i.e.*, training (1,220) and validation (305) images.

Table 1 summarizes how the images and annotations were distributed for both classification methods.

Using the categorical classification method, the trained model obtained a 94.1% validation accuracy for determining malignant tissue and its Gleason pattern. Subsequently, we evaluated the model using 100 test images that were not used during the training process. However, the concordance with our expert uropathologist analysis was only 44%. When we separately analyzed the correct prediction between groups, we found that, when the true label was benign, the model precision was 48%, whereas, for Gleason 3, 4, and 5, it was 60%, 34.6%, and 33.3%, respectively (Table 2).

**Figure 1:**
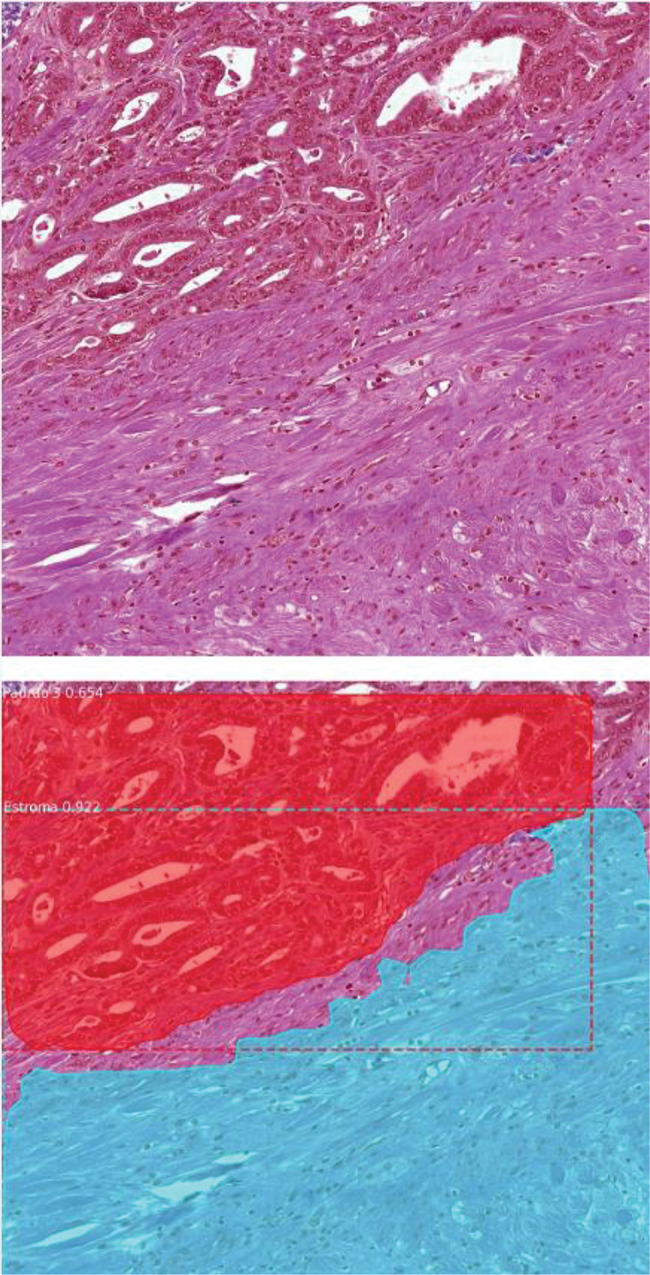
Scanning method example—The upper image shows an image patch extracted from a radical prostatectomy specimen slide. The lower image demonstrates the scanning model prediction. The method automatically detected a Gleason 3 pattern area in the upper part of the image and stroma tissue in the lower part of the patch).

With the image scanning method, our model demonstrated a validation accuracy of 91.2%. When the test images were used, the concordance between the deep learning method and uropathologists was surprisingly high; the approach correctly detected benign and cancerous tissues, including their patterns, in 89% of the never-before-seen images (Figure 1). When the annotations were evaluated individually, 117 areas were detected by the model in 100 of the images, among which 106 areas were detected correctly (90.5%) (Table 3). The correct annotation rate was as follows: 31 predictions for benign tissue (96.7% correct), 27 predictions for Gleason 3 (92.5% correct), 29 annotations for Gleason 4 (96.5% correct), and 30 predictions for Gleason 5 (76.6% correct).

## DISCUSSION

A slide analysis of a biopsy or radical prostatectomy specimen is traditionally conducted manually by pathologists, using optical microscopes. In recent years, owing to rapidly evolving visual system technologies, artificial intelligence techniques have been developed to support the work of pathologists (15).

In comparison to the results recorded by experienced uropathologists, using the proposed deep learning scanning method, we demonstrated an accuracy of 89% in real-world images in the PCa diagnosis and determination of the Gleason/ISUP grading. However, our categorical method had a low global accuracy of 44% in the never-before-seen images. These findings suggest that delimitating the areas of interest in each image patch is an extremely time-consuming and stressful activity, but can generate superior results. Using Mask-RCNN, Couteaux (16) obtained a 90.6% accuracy in automatically detecting meniscal tears in the knee, demonstrating the effectiveness of this technique and its applicability in any field of medicine.

In addition, Nagpal et al. (17) used an extremely robust database, comprising 112 million pathologist-annotated image patches from 1,226 whole-slide images, and achieved a mean accuracy of 70% compared to 61% among the 29 general pathologists. Interestingly, they reported that the tumor grading evaluations by uropathologists were significantly more accurate than those of the general pathologists (71.7% *versus* 58.0%, *p*<0.001), suggesting that the deep learning model may have a higher proficiency for tumor grading than general pathologists (18).

Litjens et al. (12) introduced deep learning as a tool for improving the objectivity and efficiency of a histopathological evaluation. They studied the deep learning performance in the PCa identification during a biopsy, and their algorithm was able to detect all slides containing PCa, whereas 30-40% of the slides containing normal tissue needed human intervention to be excluded. Using specimens from radical prostatectomies segmented in a tissue microarray, Arvaniti et al. (13) reached an inter-annotator agreement between the model and two pathologists at 0.75 and 0.71, respectively, comparable with the inter-pathologist agreement (kappa=0.71).

The accuracy in the determination of the Gleason/ISUP score depends directly on the experience of the pathologist. However, the number of pathologists in most parts of the world is insufficient for supporting the complexities of sub-specialization, which is more serious in lower-income countries such as Brazil.

Increasing the number of images is essential for improving the accuracy of our model. In addition, by evaluating the image sets, we noted that some morphologies are matter of confusion, such as the seminal vesicle epithelium and inflammatory infiltrate, which may be difficult for the algorithm to solve. We observed that, in addition to increasing the number of images, if we include different aspects of Gleason pattern 5, (*e.g.*, inflammation, atrophy, and post-atrophic hyperplasia), we believe our algorithm will be able to learn and distinguish the different morphological aspects that may be a matter of confusion.

The involvement of multiple uropathologists may also improve the quality of the image sets by selecting those achieving a consensus.

With our numbers, we want to reinforce the satisfactory results of deep learning algorithms in the diagnosis and grading of PCa, as well as their utility as a tool used in daily routines to improve quality and speed of pathologists, thereby benefiting the welfare of the society.

This is a new type of knowledge, and many variables should be assessed before excellence can be achieved. For example, what is the best machine learning method available? How many images are necessary to achieve a good agreement? Who should train the machine? Will the results be based exclusively on machine observations or will pathologists have to sign off on the final outcome? Such questions need to be addressed in future large-scale studies, which should be conducted globally.

## CONCLUSIONS

Our data have shown that a deep learning algorithm has high potential for the detection and grading of PCa. Scanning methods are likely to be superior to simple classification methods when a limited dataset is available. Future applications of deep learning methods will be unlimited, and should therefore be studied extensively during the next few years.

## AUTHOR CONTRIBUTIONS

Melo PAS and Leite KRM conceptualized the study. Melo PAS, Estivallet CLN and Leite KRM collected and analyzed the data. Melo PAS, Srougi M, Nahas WC and Leite KRM conducted the formal analysis. Melo PAS, Estivallet CLN and Leite KRM developed the methodology. Melo PAS, Estivallet CLN and Leite KRM were in charge of project administration. Melo PAS and Estivallet CLN handled the software. Melo PAS, Srougi M, Nahas WC and Leite KRM supervised the study. Melo PAS, Estivallet CLN and Leite KRM validated the data. Melo PAS, Srougi M, Nahas WC and Leite KRM visualized the study. Melo PAS, Srougi M, Nahas WC and Leite KRM wrote the manuscript.

## Figures and Tables

**Table 1:** Characteristics of annotated slides.

	n (%)
Whole prostatectomy slides	12 (100)
Categorical classification method	
Total no. of slide patches generated	1,525 (100)
Only benign tissue	740 (48.5)
Gleason 3 pattern predominant	251 (16.4)
Gleason 4 pattern predominant	254 (16.7)
Gleason 5 pattern predominant	280 (18.4)
Scanning method	
Total no. of annotations generated	1,982 (100)
**Stroma	535 (27.0)
Normal glands	559 (28.2)
Gleason 3 pattern	273 (13.8)
Gleason 4 pattern	281 (14.2)
Gleason 5 pattern	334 (16.8)

**Table 2:** Categorical classification method—true label (pathologist label) *versus* predicted label (deep learning label) in test dataset images.

		Benign	Predicted label			
			Gleason 3	Gleason 4	Gleason 5	Total
True label	Benign	12 (48%*)	2	4	7	25
	Gleason 3	4	15 (60%*)	4	2	25
	Gleason 4	4	7	9 (34.6%*)	6	26
	Gleason 5	1	6	9	8 (33.3%*)	24

* Correct concordance between pathologist analysis and trained model prediction.

**Table 3:** Scanning classification method—true area label (pathologist analysis) *versus* deep learning predicted area label in test dataset images.

		Benign	Predicted area label			
			Gleason 3	Gleason 4	Gleason 5	Total
True area label	Benign	30 (96.7%*)	1	0	6	37
	Gleason 3	1	25 (92.5%*)	1	0	27
	Gleason 4	0	1	28 (96.5%*)	1	30
	Gleason 5	0	0	0	23 (76.6%*)	23

* Correct concordance between pathologist analysis and trained model prediction.
